# Comparative Genomics of *Triticum, Secale,* and *Triticale*: Codon Usage Bias in Chloroplast Genomes and Its Implications for Evolution and Genetic Engineering

**DOI:** 10.3390/ijms262110266

**Published:** 2025-10-22

**Authors:** Tian Tian, Yinxia Zhang, Wenhua Du, Zhijun Wang

**Affiliations:** 1 College of Grassland Science, Gansu Agricultural University, Lanzhou 730070, China; ygsn2705@126.com (T.T.); 1073325010020@st.gsau.edu.cn (Y.Z.); lisang1918@126.com (Z.W.); 2 Key Laboratory of Grassland Ecosystem, Ministry of Education, Lanzhou 730070, China; 3 Pratacultural Engineering Laboratory of Gansu Province, Lanzhou 730070, China; 4 Sino-U.S. Centers for Grazingland Ecosystem Sustainability, Lanzhou 730070, China

**Keywords:** CUB of chloroplast genomes, triticale, synthetic crop, comparative genomics

## Abstract

Chloroplast codon usage bias (CUB) records both maternal phylogeny and selection intensity. Characterizing CUB in the synthetic cereal × *Triticosecale* and its Triticum and Secale parents is therefore a prerequisite for plastid-based engineering and for tracing the evolutionary consequences of recent allopolyploidy. Complete plastome sequences of five taxa—Triticum monococcum, T. turgidum, T. aestivum, Secale cereale and × Triticosecale sp.—were downloaded. Protein-coding genes were extracted to calculate overall GC, GC1–GC3, SCUO, RSCU, ENC-GC3s, neutrality, and PR2 plots. Optimal codons were defined as RSCU ≥ 1 and △RSCU ≥ 0.8. The results showed that the chloroplast genomes of these five species are low in GC content for the third base of codons, suggesting an end preference for A or U bases. The SCUO values ranged from 0.22 to 0.23, suggesting no significant codon usage bias. GC content was relatively low (38.78–39.16%), with the order GC1 > GC2 > GC3. RSCU analysis indicated that codons ending with A/T are more commonly used. Neutral mapping, ENC-GC3s, and the PR2 plot all showed that the preference of codon usage for the majority of functional genes was influenced by a combination of mutation and natural selection pressure, and the influence of natural selection was predominant. RSCU clustering recovers the expected maternal tree (Triticum clade + triticale). All optimal codons terminate with A or U, yielding identical plastid translation tables for the five species. Despite its recent hybrid origin, triticale plastid CUB is indistinguishable from its wheat maternal ancestor and is governed mainly by selection. The compiled optimal codon set provides an immediate reference for chloroplast transformation and for dissecting selection relaxation in newly synthesized triticale combinations.

## 1. Introduction

Chloroplasts are semi-autonomous organelles that convert solar energy into biological energy through photosynthesis and synthesize many other compounds [1,2,3,4]. The chloroplast genome is essential for these active metabolic functions [3,4]. Detailed characterization of individual chloroplast genomes and comparative chloroplast genomics can underpin functional genomic research and guide chloroplast genetic engineering experiments [5,6,7]. The chloroplast genome can also be used to develop molecular markers for phylogenetic analysis, population-level studies, genotyping, and gene mapping [8]. Chloroplast gene transformation has several advantages, such as high-efficiency expression of foreign genes, stable inheritance, site-specific integration, and no position effect [9,10]. The codon is crucial for translating genetic information into proteins. Among the 64 codons, 61 encode the 20 standard amino acids, with 3 being stop codons. While tryptophan and methionine are each encoded by a single codon, the remaining 18 amino acids are usually encoded by multiple synonymous codons. Research indicates that synonymous codon usage is not random, leading to synonymous codon usage bias (SCUB) [11,12,13]. SCUB is influenced by base mutations, environmental factors, genetic drift during evolution, and gene expression levels, with natural selection and mutation being the main factors. It also serves as a way to fine-tune gene expression [12,14,15,16]. Studying codon usage bias can optimize codon usage and promote research on species evolution. SCUB is common across different species, tissues, and genes, and studying it can enhance the expression efficiency of foreign sequences in transgenic research. It is also significant for gene expression regulation, genome evolution, species evolution studies, and bioinformatic research [17,18].

Wheat (*Triticum* spp.) is one of the most widely cultivated crops globally. It accounts for about 17% of the world’s cultivated land and accounts for 20% of the world’s food consumption. This crop is highly valued for its high starch and protein content and its high yield [19,20]. Rye (*Secale cereale* L.), known for its robust root system, strong stress resistance, and rich nutritional profile, has a protein content of approximately 17%. Its amino acid composition generally exceeds that of common wheat, making it a valuable fodder crop [21].

**Figure 1:**
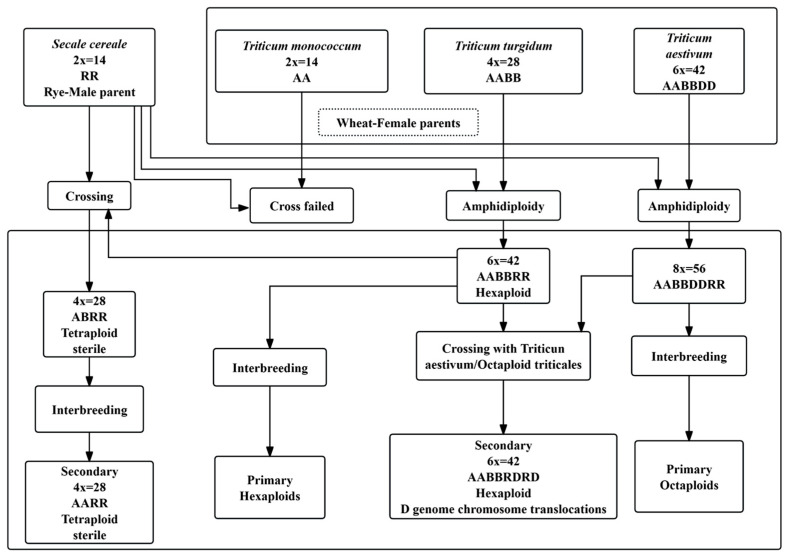
Summary chart of the origins of different types of triticale.

Triticale (× *Triticosecale Wittm*), a man-made crop, is a hybrid derived from crosses between wheat species (*Triticum monococcum*, *Triticum turgidum* and *Triticum aestivum*) and rye (*Secale cereale*), followed by chromosome doubling [22,23]. It combines the high yield and superior quality of wheat with the disease and stress resistance of rye and its high lysine content, showing significant hybrid vigor. In the Northwest of China, triticale has become a high-quality forage crop after alfalfa and corn [24,25]. Currently, triticale mainly exists in two types (Figure 1): hexaploid (AABBRR) and octoploid (AABBDDRR) [26,27]. Hexaploid triticale (2n = 6x = 42, AABBRR) can be synthesized through two alternative pathways [28]: (i) by crossing tetraploid wheat (AABB, 2n = 4x = 28) with rye (RR, 2n = 2x = 14) and subsequently doubling the chromosomes of the sterile F1 hybrid (ABR, 2n = 21), yielding a new amphidiploid carrying 21 chromosome pairs, 7 of which are derived from rye; (ii) by crossing hexaploid wheat (AABBDD, 2n = 6x = 42) with rye (RR), with the initial hybrid in this route possessing the full AABBDD RR complement, but hexaploid triticale being recovered after several generations of selection for progenies that have eliminated the entire D genome. Octoploid triticale (AABBDDRR) is formed by doubling the chromosomes of the F1 hybrid from hexaploid wheat (AABBDD) and rye. Studies have shown that triticale has good salt and alkali tolerance, capable of growing in saline–alkali soils with a salinity of 4‰ to 12‰, making it a high-quality genetic resource for the biological improvement of saline–alkali soils. Therefore, breeding high-yielding, disease- and stress-resistant triticale has always been the goal of genetic improvement. However, this is limited by triticale’s complex genetic background, polyploidy, and the long cycle of traditional breeding. Molecular breeding can alleviate these problems to some extent. But the expression level of foreign genes is unstable and can be affected by codon usage bias [29]. Codon usage bias has been widely studied in chloroplast genomes, partly due to the advantages of chloroplast genomes in species evolution research. As a synthetic crop established only about 140 years ago, triticale has not undergone long-term natural divergence, so its plastid genome strictly mirrors the maternal wheat lineage. Thus, codon usage patterns of the chloroplast genome in triticale accessions can be used to trace the maternal wheat lineage that contributed to the initial synthetic crosses.

Here we report a comparative analysis of chloroplast genome-wide codon usage across five representative taxa: *Triticum monococcum*, *T. turgidum*, *T. aestivum*, *Secale cereale* and × *Triticosecale* sp. Then, we analyzed and compared the CUB of these species, including GC, GC1–GC3, SCUO, RSCU, ENC-GC3s, neutrality, PR2 plots, and optimal codons. These data were then used to: (i) clarify the maternal origin of the triticale plastome by determining which wheat lineage clusters with triticale; (ii) test whether artificial allopolyploidy has relaxed selective constraints acting on the plastid genome constraints, by comparing the strength and direction of codon bias between synthetic triticale and its natural wheat donors.

## 2. Results

### 2.1. Nucleotide Composition Analysis of cp Genes in Triticum, Secale, and Triticale

**Figure 2:**
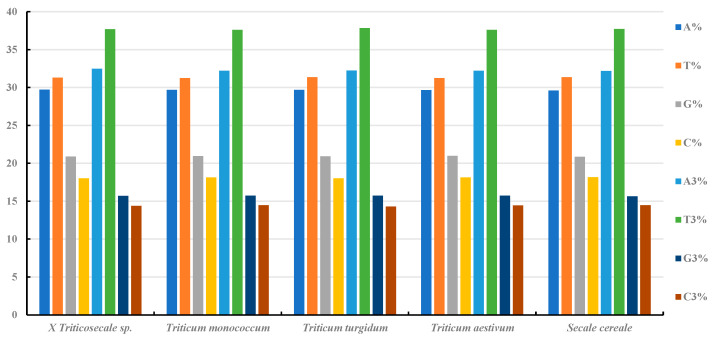
Distribution of nucleotides for cp genes in *Triticum*, *Secale*, and *Triticale*. Unequal distribution of nucleotide composition was observed.

**Figure 3:**
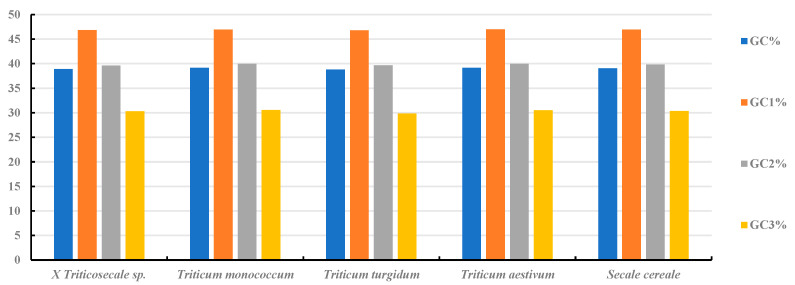
Distribution of overall GC content, GC1, GC2, and GC3 of cp genes in *Triticum*, *Secale*, and *Triticale*. The overall GC content was lower than 50%, and the greatest difference in GC content was observed between GC1 and GC3.

In the chloroplast genomes of *Secale*, *Triticum*, and *triticale*, A/T bases are predominant, with the T base showing the highest frequency. At the third codon position, A3 and T3 are most prevalent, and G3 also exhibits a high frequency, indicating an A/T bias in all five species studied (Figure 2). The SCUO values for these species range from 0.22 to 0.23, suggesting no significant codon usage bias (Table S1). In all five species, the GC content is uniformly low (38.78~39.16%), with the order GC1 > GC2 > GC3, highlighting uneven codon distribution and a preference for A/U at the third codon position. Notably, GC3 is below 50% in all species, further confirming the A/U bias (Figure 3). The MILC values also indicate a strong codon usage bias in these species. These findings are consistent with the hypothesis that codons in higher-plant chloroplasts tend to end with A/U, indicating a conserved pattern of codon usage during evolution. Regression analysis of GC1, GC2, and GC3 revealed no significant correlation between GC1 and GC3 (regression coefficients of 0.24~0.29) or between GC2 and GC3 (regression coefficients of −0.055~0.128), suggesting high GC=content conservation and limited mutation in these species.

### 2.2. Relative Synonymous Codon Usage (RSCU) Analysis of Triticum, Secale, and Triticale

**Figure 4:**
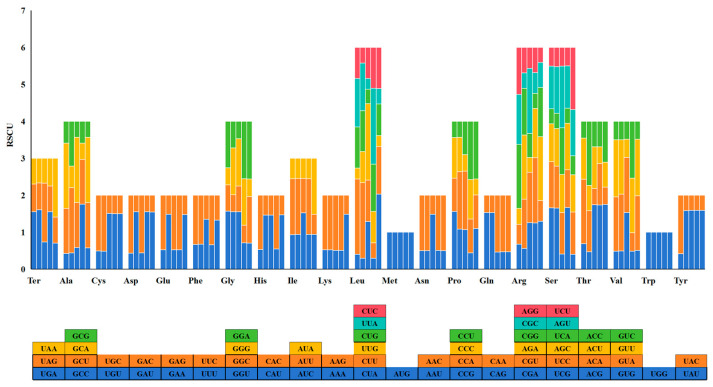
RSCU analysis of codons encoding amino acids in the chloroplast genomes of *Triticum*, *Secale*, and *Triticale*. Note: The species from left to right are × *Triticosecale* sp., *Triticum monococcum*, *Triticum turgidum*, *Triticum aestivum*, and *Secale cereale*. The square below represents all codons encoding each amino acid, and the height of the bar above represents the sum of all codon RSCU values.

An analysis of codon usage frequency in five species revealed that, among the 59 codons, excluding AUG, UGG, UGA, UAA, and UAG, 30 end with A/T and 29 with G/C (Figure 4). Codons ending with A/T were found to be dominant. Furthermore, five codons (UUA, GCU, ACU, AGA, and UCU) were over-represented (RSCU > 1.6) in all five species (Table S2), indicating a preference for these codons in their chloroplast genomes.

### 2.3. Mutational Pressure Versus Natural Selection in CUB of cp Genes of Triticum, Secale, and Triticale

We analyzed the relationship between overall nucleotide content and third-codon-position nucleotide content across the five species to explore codon usage patterns (Table 1). Strong positive correlations were detected between the genomic frequencies of homologous bases and their third-position frequencies (e.g., A vs. A3, T vs. T3), whereas most heterologous pairs (e.g., A vs. T3, G vs. C3) exhibited significant negative or no correlation. Likewise, GC content was highly positively correlated with GC3 in all species. These patterns suggest that third-position composition is conserved in parallel with overall genomic composition, and that natural selection, rather than mutational bias, is the dominant force shaping codon usage.

### 2.4. Inter-Relationship of CUB and Nucleotide Composition

We assessed the codon preference of chloroplast genomes in five species using the SCUO value. As shown in Table 2, the average SCUO values of these species were all below 0.5, indicating weak codon preference. Correlation analysis between SCUO and codon composition revealed that SCUO values were significantly negatively correlated with the G base in all species (*p* < 0.05). In Triticum aestivum, SCUO values were also significantly negatively correlated with G3 (*p* < 0.05). This suggests that the G base content in all species influences codon preference, and in Triticum aestivum, codon preference is shaped by both the G base and G3.

### 2.5. Relationship Between Codon Usage Bias and Gene Expression

The MILC value is positively correlated with the level of gene expression (Table S3). Our calculations revealed MILC values between 0.6 and 0.8 for all five species, indicating relatively high gene expression levels. Further analysis identified the top five highly expressed genes across these species. Notably, the *rps*8 and *ndh*G genes exhibited high expression levels in all species. The *ycf*68 gene was highly expressed in × *Triticosecale* sp., *Triticum monococcum*, *Triticum aestivum*, and *Secale cereale*. The *rpl*16 gene showed high expression in all species except *Triticum monococcum*, and the *rpl*20 gene was highly expressed in × *Triticosecale* sp., *Triticum monococcum*, and *Triticum turgidum*. Conversely, the genes with the lowest expression levels across all species were *psb*B, *rps*3, *psb*D, *rbc*L, and *psb*A.

### 2.6. ENC-Plot Analysis

**Figure 5:**
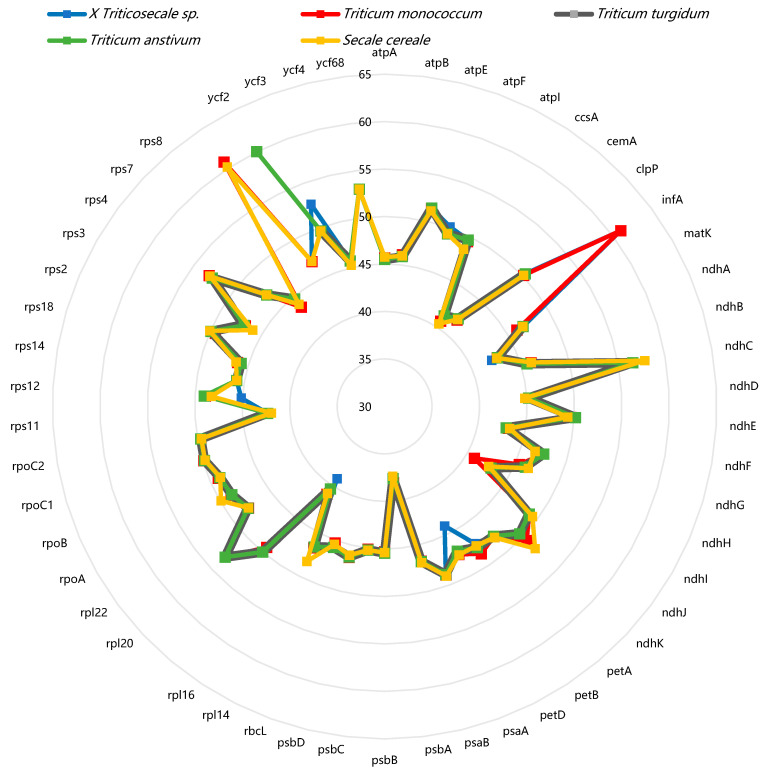
The ENC-plot analysis in *Triticum*, *Secale*, and *Triticale*.

**Figure 6:**
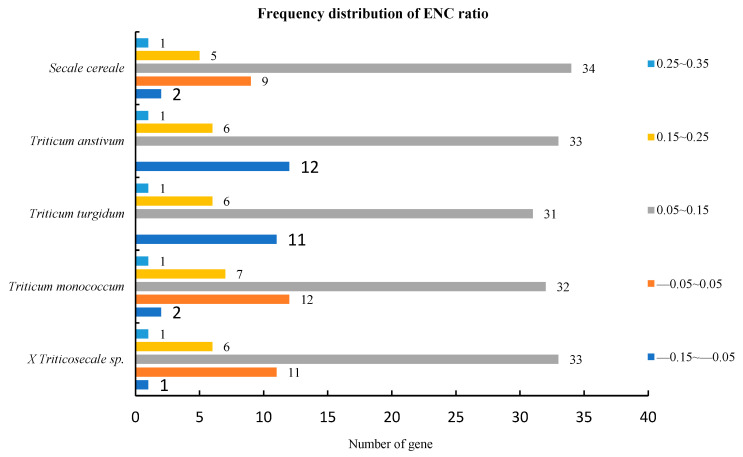
Frequency distribution of ENC ratio in chloroplast genes in *Triticum*, *Secale*, and *Triticale*.

Based on ENC bias analysis (Figure 5), the ENC values for × *Triticosecale* sp., *Triticum monococcum*, *Triticum turgidum*, *Triticum aestivum*, and *Secale cereale* ranged from 61 to 37.64, 61 to 38.08, 56.55 to 37.64, 60.04 to 37.64, and 60.18 to 37.4, respectively. The average values were 47.31, 47.66, 47.05, 47.45, and 47.31. Genes with ENC > 45 accounted for 42, 45, 40, 43, and 41 in each species, indicating relatively weak codon usage bias. To better understand this trend, we calculated the ENC-ratio frequency distribution, using the range of −0.05~0.05 as a threshold for quantifying deviations from expected values (Figure 6). The results showed that in all species, few genes fell within this range: 11 (21.15%), 12 (22.22%), 11 (22.45%), 12 (23.08%), and 9 (17.65%) of the genes in × *Triticosecale* sp., *Triticum monococcum*, *Triticum turgidum*, *Triticum aestivum*, and *Secale cereale*, respectively. Most genes lay outside this range: 41 (78.85%), 42 (77.78%), 38 (77.55%), 40 (76.92%), and 42 (82.35%) of the genes in × *Triticosecale* sp., *Triticum monococcum*, *Triticum turgidum*, *Triticum aestivum*, and *Secale cereale*, respectively. This suggests that natural selection has a greater influence on codon usage bias than mutation in these species.

### 2.7. PR2-Bias-Plot Analysis

**Figure 7:**
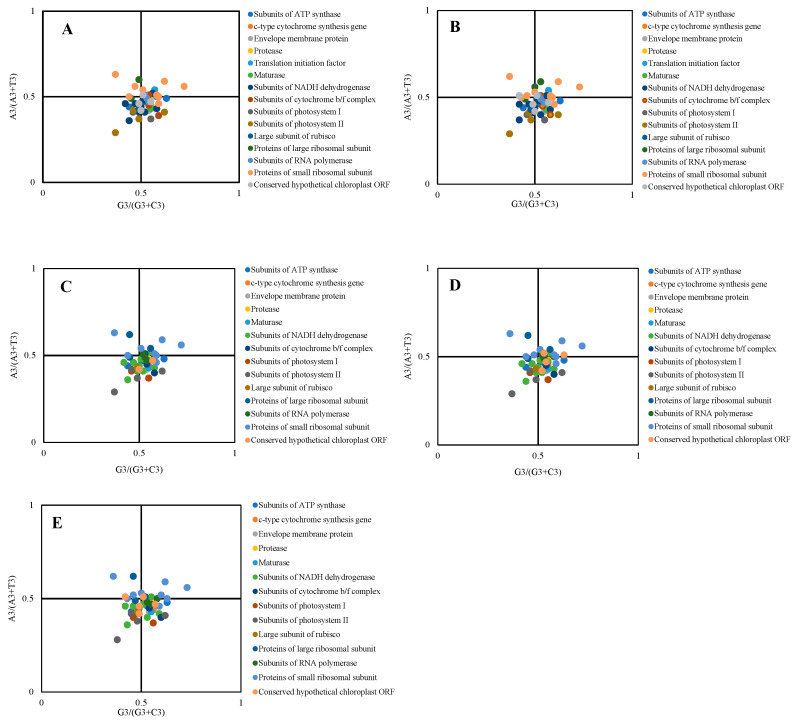
PR2-bias plot of cp genes in different species of *Triticum, Secale*, *and Triticale*. (**A**): × *Triticosecale* sp.; (**B**): *Triticum monococcum*; (**C**): *Triticum turgidum*; (**D**): *Triticum aestivum*; (**E**): *Secale cereale*. A and T were not equal to G and C in fourfold degenerate codons.

PR2-plot analysis reveals that when mutation is the sole influence, the four bases are evenly distributed, clustering around the center point. However, when selection pressure is also at play, the data points deviate from the center to varying extents. In our analysis of the five species, most genes were found below the center point, particularly in the lower-right section (Figure 7). This indicates a base usage frequency of G3 > C3 and T3 > A3. Genes related to Subunits of photosystem I, Subunits of photosystem II, and Large subunit of rubisco were mostly located in the lower left, suggesting a preference for C3 > G3 and T3 > A3. The genes were unevenly distributed across the figure, with the highest density in the bottom-left and bottom-right areas and only sparse presence elsewhere. This implies that base usage is influenced by both mutation and selection.

The five species exhibit broadly similar gene distribution trends in the PR2-plot analysis. However, Triticum monococcum shows subtle differences, with most of its genes, such as those related to Subunits of RNA polymerase and Proteins of large ribosomal subunit, clustering closer to the center point. In contrast, × *Triticosecale* sp., *Triticum turgidum*, *Triticum aestivum*, and *Secale cereale* display more similar gene distributions. Notably, two genes with functions of Conserved hypothetical chloroplast ORF and Subunits of RNA polymerase are almost near the center point, indicating a weaker bias and greater influence of mutation on their codon usage. Overall, most genes were found away from the center point, indicating that natural selection has a greater impact on codon usage bias than mutation.

### 2.8. Neutrality Plot

**Figure 8:**
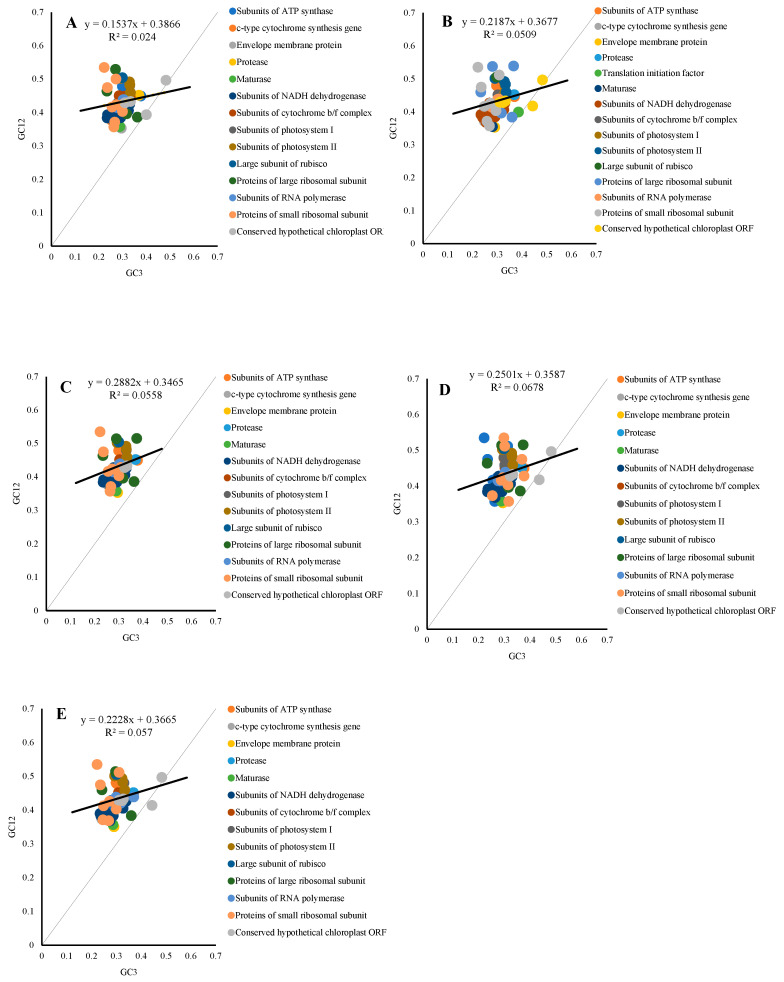
Neutrality plot of cp genes in different species of Triticum, Secale, and Triticale. (**A**): × *Triticosecale* sp.; (**B**): *Triticum monococcum*; (**C**): *Triticum turgidum*; (**D**): *Triticum aestivum*; (**E**): *Secale cereale*. (Black line represents the regression trend of gene distribution. The angle formed between the extension of this trend line and the neutral reference line (y = x) reflects the relative intensity of mutational pressure versus natural selection acting on codon usage bias. A larger angle indicates a stronger influence of mutational pressure, while a smaller angle suggests that natural selection plays a more dominant role).

We used neutrality plots to determine the main factor influencing codon usage bias in the chloroplast genomes of five species. In our results (Figure 8), all species showed weak and non-significant correlations between GC3 and GC12, indicating that natural selection was the main factor influencing codon usage bias. In the plot, apart from Triticum turgidum, one gene each from × *Triticosecale* sp., *Triticum monococcum*, *Triticum aestivum*, and *Secale cereale* was located below the diagonal line, with the rest above it. This further indicated that natural selection was the primary influence. Additionally, three genes related to the functions of Conserved hypothetical chloroplast ORF and Proteins of large ribosomal subunit in × *Triticosecale* sp., *Triticum aestivum*, *and Secale cereale*, and four genes related to Envelope membrane protein, Translation initiation factor, and Conserved hypothetical chloroplast ORF in *Triticum monococcum*, were found near or below the diagonal line, suggesting that some genes were more influenced by mutation.

Neutrality plot analysis can further be used to explain the role of mutation pressure and natural selection. Natural selection is dominant when the slope of the regression line is close to 0, whereas, if the slope of the regression line is close to 1, then mutation pressure is said to play the major role [30,31]. The regression line slopes for × *Triticosecale* sp., *Triticum monococcum*, *Triticum turgidum*, *Triticum aestivum*, and *Secale cereale* were 0.15, 0.22, 0.29, 0.25, and 0.22, respectively. This further indicated that natural selection was the primary factor shaping codon usage bias in these species.

### 2.9. Optimal Codons

By constructing high- and low-expression gene libraries and selecting codons with ΔRSCU ≥ 0.08 and RSCU ≥ 1, we identified optimal codons for each species (Table 3). We found 16, 17, 13, 18, and 18 optimal codons in × *Triticosecale* sp., *Triticum monococcum*, *Triticum turgidum*, *Triticum aestivum*, and *Secale cereale*, respectively. All optimal codons ended with A/U, consistent with the GC3 and RSCU analysis results.

### 2.10. Phylogenetic Relationships Between Triticale and Its Parental Species Were Inferred from the RSCU Values

**Figure 9:**
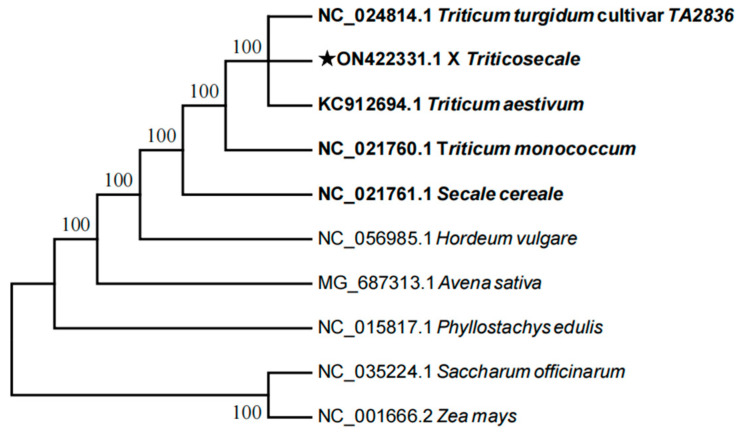
Phylogenetic trees of *Triticum*, *Secale*, and *Triticale* constructed using chloroplast genome sequences. (The ★ denotes Triticale, the hybrid of wheat and rye, and is used for emphasis).

**Figure 10:**
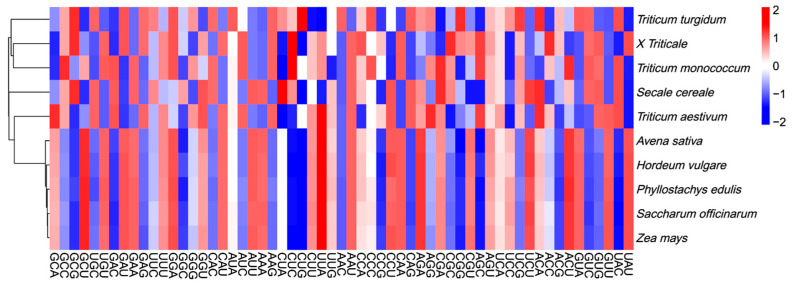
Cluster analysis of *Triticum*, *Secale*, and *Triticale* constructed using RSCU.

Phylogenetic trees were constructed for five species (× *Triticosecale* sp., *Triticum monococcum*, *Triticum turgidum*, *Triticum aestivum*, and *Secale cereale*) along with five outgroups (*Hordeum vulgare* L., *Avena sativa* L., *Saccharum officinarum* L., *Zea mays* L., *Phyllostachys edulis* (Carrière) J. Houz.) using the Maximum-Likelihood method and Minimum-Evolution Tree model, based on whole chloroplast genome sequences (Figure 9) and RSCU values (Figure 10). The results showed that both clustering methods produced similar topologies. Triticale showed a close affinity to *Triticum turgidum* and *Triticum aestivum*. In the RSCU-based tree, the five outgroups formed a separate cluster closely related to *Secale cereale* and *Triticum aestivum*. Triticale clustered with *Triticum turgidum* and *Triticum monococcum*, indicating a close relationship. Overall, the phylogenetic trees derived from both whole chloroplast genome sequences and RSCU values showed consistent evolutionary relationships, suggesting that RSCU values can effectively reflect phylogenetic distances and are useful for assessing species relatedness.

## 3. Discussion

Codon usage bias is a crucial feature in genome evolution. The frequency of codon usage unveils evolutionary trends, as it is jointly determined by natural selection and mutation. The “selection–mutation–drift” hypothesis posits that codon bias arises from selection pressure for optimal codons and non-synonymous codon “mutation–drift”, whereas the “neutral theory” hypothesis views synonymous codon mutations as neutral, unrelated to selection pressure [32,33]. Various factors such as GC content, neighboring nucleotide composition, population size, and gene expression levels also influence codon usage bias [34]. This bias can affect gene expression by influencing mRNA folding, translation elongation, and protein folding [35]. These factors are widely studied in genomic research [36,37]. Previous studies indicate that lower plants tend to use G/C-ending codons, while higher plants favor A/U-ending codons [38]. In our study, the five species have low GC content and a preference for A/U-ending codons, consistent with the theory. This suggests that these genomes may have reached an evolutionary equilibrium over time.

In most plants, A/U bases are common at the third codon position, mainly due to natural selection. For example, in 18 Oryza species (Poaceae family), many codons end with A/U, showing selection-driven codon usage bias [34]. Our correlation analysis of codon composition in five species revealed that homologous nucleotides (A/A3, T/T3, G/G3, C/C3) are highly positively correlated, while most heterologous nucleotides show significant negative or no correlation. GC and GC3 are also highly positively correlated. These results demonstrate that base composition is highly conserved and that codon usage bias is primarily shaped by natural selection.

The SCUO value is a measure of the degree of codon usage bias. In our study, the SCUO values for all species were found to be low (all <0.25), indicating weak codon usage bias. This is consistent with previous findings in Pisum species such as *Pisum fulvum* Sm. and *Pisum sativum* L. [39]. Additionally, the MILC value, which can reflect the level of gene expression, was found to be high in the five species studied. This suggests that gene expression is favorable in these species, which may be related to the conservation of codon usage. The low SCUO values indicate weak codon bias but strong conservation, implying that codon usage is less prone to mutation, thereby stabilizing the expression of certain genes.

The correlation analysis of GC12 and GC_3_ shows that natural selection is the main factor influencing the chloroplast codon usage bias of the five species. The regression line slope indicates that codon usage bias is jointly affected by natural selection and mutation, but mainly by natural selection. In the PR2-plot analysis, it was found that in all species, the codon usage bias of individual genes is more influenced by mutation. At the same time, the frequency of codon usage is somewhat correlated with gene function. Most genes are located in the lower right of the plot, with an obvious usage preference of G3 > C3 and T3 > A3, which further indicates the preference for U-ending codons in the five species. The size of the ENC value is negatively correlated with the strength of the species’ codon usage bias [13]. The results show that the codon usage bias in the five species is relatively weak, and natural selection is the main influencing factor.

Phylogenetic analysis shows that the five species studied have a conserved evolutionary history. Triticale shows a closer genetic relationship to its female parent, indicating a more intimate phylogenetic affinity. This finding furnishes a theoretical directive for subsequent breeding endeavors: by exploiting the propensity for maternal inheritance, we may designate plants bearing the desired trait as the maternal parent during hybridization. And based on RSCU values, the clustering analysis aligns with the results from chloroplast genome sequence-based clustering, suggesting that RSCU-based clustering can be used to assess species relatedness.

We identified optimal codons for each of the five species based on RSCU values. These optimal codons can enhance or optimize gene expression in heterologous systems, which is crucial for genetic engineering, especially in transgenic crop breeding [40,41]. Our analysis of optimal codons in the five species (× *Triticosecale* sp., *Triticum monococcum*, *Triticum turgidum*, *Triticum aestivum*, and *Secale cereale*) revealed 16, 17, 13, 18, and 18 optimal codons, respectively. Triticale’s optimal codons closely resemble those of *Triticum monococcum*, *Triticum aestivum*, and *Secale cereale* in both number and type, indicating that triticale has inherited more from its female parent. And identifying optimal codons provides a theoretical basis for enhancing foreign gene expression levels and understanding gene regulation mechanisms in *Triticum*, *Secale*, and *triticale species*.

## 4. Materials and Methods

### 4.1. Sequence Retrieval

The complete chloroplast genome sequences of the five species were downloaded from the NCBI database (https://www.ncbi.nlm.nih.gov (accessed on 26 April 2025)). For × *Triticosecale* sp. (GenBank accession number: ON422331.1), 87 sequences were obtained; for *Triticum monococcum* (NC_021760.1), 79 sequences; for *Triticum turgidum* (NC_024814.1), 82 sequences; for *Triticum aestivum* (KC912694.1), 79 sequences; and for *Secale cereale* (NC_021761.1), 77 sequences. After filtering out sequences shorter than 300 bp, removing duplicates, excluding sequences without ATG as the start codon, and eliminating sequences with abnormal termination codons or internal termination codons within the CDS, the final datasets comprised 52, 54, 49, 52, and 51 sequences, respectively, for subsequent analyses.

### 4.2. Analysis of Codon Composition

CodonW 1.4.2 was used to calculate the codon adaptation index (CAI) for the filtered chloroplast genome CDS sequences of the five species. The online software CUSP (http://emboss.toulouse.inra.fr/cgibin/emboss/cusp (accessed on 28 April 2025)) was employed to determine the codon count (CN) and GC content at each codon position (GC1, GC2, GC3) and overall GC content (GC) for these sequences. The average of GC1 and GC2 (GC12) was also calculated, and correlation analyses were performed using the R version 4.4.2.

### 4.3. Synonymous Codon Usage Order (SCUO) Analysis

The SCUO value can assess chloroplast genome codon usage bias and is calculated as shown below.

Codon usage entropy for the nth amino acid:

$$H_{i}=-\sum_{j=1}^{ni}p_{\mathrm{ij}}\log_{2}p_{\mathrm{ij}}$$

Here, *H*_i_ is the codon usage entropy for the ith amino acid, *n_i_* is the number of synonymous codons, and *p_ij_* is the usage frequency of the ith codon for the *j*th amino acid.

SCUO value for the nth amino acid:

$$SCUO_{i}=1-\frac{Hi}{Hi,\max}$$

Here, *SCUO_i_* is the SCUO value for the nth amino acid, and *H_i,max_* is the ith codon usage entropy when all synonymous codons are used randomly. It is calculated as

$$H_{i,\max}=\log_{2}n_{i}$$

Average SCUO value for the entire sequence:

$$SCUO=\frac{1}{N}\sum_{i=1}^{n}SCUO_{i}$$

Here, *SCUO* is the average SCUO value for the entire sequence, and *n* is the total number of amino acids in the chloroplast genome.

### 4.4. Relative Synonymous Codon Usage (RSCU) Analysis and Measure Independent of Length and Composition (MILC)

Relative synonymous codon usage (RSCU) is the ratio of a codon’s actual usage frequency to its expected frequency. An RSCU value of 1 indicates no usage preference; a value greater than 1 suggests higher-than-expected usage; and a value less than 1 indicates lower usage. The RSCU value for the *j*th codon of the *i*th amino acid is calculated as follows:

$$RSCU_{ij}=\frac{X_{\mathrm{ij}}}{\frac{1}{n_{i}}\sum_{j=1}^{ni}X_{\mathrm{ij}}}$$

Here, *X_ij_* is the frequency of occurrence of the *j*th codon for ith amino acid (any *X_ij_* with a value of zero is arbitrarily assigned a value of 0.5), and n_i_ is the number of codons for the ith amino acid (ith codon family).

The MILC (Measure Independent of Length and Composition) value is positively correlated with gene expression levels. It is a measure of gene expression that is independent of gene length and nucleotide composition. A higher MILC value indicates a higher level of gene expression, and vice versa.

It is calculated as

$$MILC_{i}=1-CAI_{i}$$

Here, *MILC_i_* is the MILC of the ith codon, and *CAI_i_* is the adaptation index for that codon of the ith codon.

### 4.5. PR-2 Plot

To understand the impact of mutation and selection pressure on the codon usage of cp genes, the Parity rule 2 (PR2)-plot analysis was performed. In this analysis, A3/(A3 + T3) was plotted on the *y*-axis and G3/(G3 + C3) on the *x*-axis. Each gene’s base composition was represented on a planar plot. The center point (0.5, 0.5) represents no codon usage bias (A=T and G=C). Points deviating from the center indicate the direction and extent of bias. Genes near the center line or close to the center point indicate that codon usage is mainly influenced by mutation; those scattered around or far from the center are influenced by both mutation and natural selection [42].

### 4.6. Neutrality Plot Analysis

A neutrality plot was constructed with GC3 on the *x*-axis and the average of GC1 and GC2 (GC12) on the *y*-axis. Genes aligning along the diagonal are predominantly influenced by mutation, whereas those clustered around the diagonal are more affected by selection pressure. The correlation between GC3 and GC12 determines the influence of mutation or selection: a significant correlation with a regression coefficient close to 1 indicates that mutation is the main factor, while a coefficient near 0 suggests that selection pressure is dominant [43].

### 4.7. ENC-Plot Analysis

ENC measures synonymous codon usage bias. An ENC value of 20 indicates that each amino acid uses only one codon (extreme bias). A value of 61 suggests random codon usage with no bias. We constructed a standard curve (expected value), with each point on it representing a gene, to analyze the relationship between ENC values and GC3 content.

ENC is calculated as

$$ENC=\frac{2+GC_{3}+29}{{GC_{3}}^{2}+{\left(1-GC_{3}\right)}^{2}}$$

The distance between each gene and the standard curve (expected value) serves as a criterion for determining the factors influencing codon bias. When the actual ENC value significantly deviates from the expected value (i.e., the gene is far from the standard curve), codon bias is primarily influenced by selection pressure. Conversely, when the actual ENC value is close to the expected value (i.e., the gene is near the standard curve), codon bias is mainly shaped by mutation. To better reflect the difference between actual and expected ENC values, the frequency distribution of ENC ratios can be calculated, with differences quantified within the range of −0.05 to 0.05. Within this range, codon bias is largely mutation-driven; outside this range, selection pressure is the dominant factor.

### 4.8. Statistical Analysis

All statistical analyses were carried out using the software SPSS 25.0 for Windows.

## 5. Conclusions

Comparative analysis of codon usage bias in the chloroplast genomes of × *Triticosecale* sp., *Triticum monococcum*, *Triticum turgidum*, *Triticum aestivum*, and *Secale cereale* reveals a preference for A/U-ending codons in all five species. Codon usage bias is shaped by both natural selection and mutation, with natural selection being the dominant force. Triticale shows a closer codon usage pattern to its female parents, *Triticum turgidum* and *Triticum aestivum*, which is supported by phylogenetic analysis. Clustering based on RSCU values aligns well with the species’ phylogenetic relationships, indicating that RSCU values can be used to identify the affinities of species.

## Figures and Tables

**Table 1:** Correlation coefficients between overall nucleotide composition content and its composition at third codon position for cp genes in *Triticum*, *Secale*, and *Triticale*.

Species Name		A3	T3	G3	C3	GC3
× *Triticosecale* sp.	A	0.734 **	0.071	−0.144	−0.225	0.008
	T	−0.033	0.728 **	−0.072	−0.133	−0.067
	G	0.164	−0.008	0.373 **	−0.272 *	−0.311 *
	C	−0.102	0.016	−0.333 **	0.374 **	−0.088
	GC	0.098	−0.278 *	0.242	−0.041	0.542 **
*Triticum monococcum*	A	0.737 **	0.07	−0.146	−0.226	0.093
	T	−0.031	0.727 **	−0.075	−0.132	−0.104
	G	0.164	−0.005	0.373 **	−0.272 *	−0.213
	C	−0.104	0.009	−0.335 **	0.381 **	−0.121
	GC	−0.06	−0.166	0.26	−0.132	0.606 **
*Triticum turgidum*	A	0.738 **	0.068	−0.138	−0.224	0.2
	T	−0.028	0.729 **	−0.052	−0.14	0.058
	G	0.164	−0.001	0.381 **	−0.270 *	−0.177
	C	−0.098	0.015	−0.334 **	0.368 **	−0.13
	GC	0.029	−0.184	0.28	−0.073	0.554 **
*Triticum aestivum*	A	0.738 **	0.066	−0.146	−0.225	0.109
	T	−0.03	0.728 **	−0.073	−0.137	−0.181
	G	0.163	−0.005	0.377 **	−0.271 *	−0.202
	C	−0.103	0.012	−0.334 **	0.378 **	0.173
	GC	0.044	−0.214	0.226	−0.006	0.629 **
*Secale cereale*	A	0.737 **	0.071	−0.149	−0.226	0.094
	T	−0.031	0.729 **	−0.073	−0.13	−0.081
	G	0.166	−0.01	0.375 **	−0.270 *	−0.131
	C	−0.099	0.014	−0.334 **	0.377 **	−0.151
	GC	0.101	−0.071	0.05	−0.255	0.624 **

Note: * significant at *p* < 0.05 (two-tailed); ** significant at *p* < 0.01 level (two-tailed).

**Table 2:** Correlation coefficients between SCUO and compositional features of cp gene in *Triticum*, *Secale*, and *Triticale*.

Species Name		A	T	G	C	GC	A3	T3	G3	C3	GC3
× *Triticosecale* sp.	SCUO	0.187	−0.16	−0.484 *	0.229	−0.022	−0.144	0.178	−0.441	0.051	−0.221
	P	0.458	0.526	0.042	0.361	0.93	0.569	0.48	0.067	0.842	0.377
*Triticum monococcum*	SCUO	0.208	−0.175	−0.494 *	0.221	−0.031	−0.121	0.159	−0.466	0.034	−0.234
	P	0.407	0.487	0.037	0.378	0.902	0.632	0.529	0.051	0.894	0.35
*Triticum turgidum*	SCUO	0.2	−0.157	−0.497 *	0.225	−0.042	−0.127	0.158	−0.447	0.038	−0.2
	P	0.426	0.534	0.036	0.368	0.87	0.614	0.532	0.063	0.88	0.426
*Triticum aestivum*	SCUO	0.292	−0.136	−0.526 *	0.07	−0.16	−0.136	0.187	−0.543 *	0.002	−0.333
	P	0.24	0.591	0.025	0.781	0.526	0.592	0.457	0.02	0.994	0.176
*Secale cereale*	SCUO	0.219	−0.18	−0.493 *	0.244	−0.037	−0.125	0.158	−0.457	0.042	−0.211
	P	0.383	0.474	0.038	0.329	0.884	0.621	0.532	0.057	0.868	0.4

* Significant at *p* < 0.05 level (two-tailed).

**Table 3:** Optimal codon analysis in chloroplast genomes in *Triticum*, *Secale*, *and Triticale*.

× *Triticosecale* sp.			*Triticum monococcum*			*Triticum turgidum*			*Triticum aestivum*			*Secale cereale*		
AA	Codon	ΔRSCU	AA	Codon	ΔRSCU	AA	Codon	ΔRSCU	AA	Codon	ΔRSCU	AA	Codon	ΔRSCU
Ala	GCU ***	0.81	Ala	GCU ***	0.79	Ala	GCA *	0.13	Ala	GCU ***	0.6	Ala	GCA *	0.23
Arg	AGA **	0.36	Arg	CGU ***	1.16	Ala	GCU ***	0.66	Arg	AGA *	0.21	Ala	GCU ***	0.57
Arg	CGU ***	1.43	Asp	GAU *	0.24	Arg	AGA **	0.33	Arg	CGU ***	1.1	Arg	AGA *	0.23
Gln	CAA *	0.08	Cys	UGU ***	1.04	Arg	CGU ***	0.9	Asp	GAU *	0.16	Arg	CGU ***	1.14
Gly	GGU ***	0.89	Gln	CAA *	0.12	Cys	UGU ***	0.5	Cys	UGU ***	0.6	Asp	GAU *	0.19
His	CAU *	0.18	Gly	GGU ***	0.79	Gly	GGU **	0.36	Gln	CAA *	0.27	Cys	UGU **	0.42
Ile	AUU *	0.27	Ile	AUU **	0.42	Leu	CUA ***	0.67	Gly	GGU ***	1	Gln	CAA *	0.1
Leu	UUA ***	1.56	Leu	CUU *	0.1	Leu	UUA ***	0.6	His	CAU *	0.11	Gly	GGU ***	1.06
Lys	AAA ***	0.6	Leu	UUA ***	1.07	Lys	AAA *	0.09	Ile	AUU **	0.44	His	CAU *	0.15
Phe	UUU *	0.14	Lys	AAA *	0.26	Pro	CCU ***	1.09	Leu	CUA *	0.18	Ile	AUU **	0.31
Pro	CCU ***	1.22	Pro	CCA *	0.18	Ser	UCU ***	1.19	Leu	UUA ***	1.16	Leu	CUU *	0.21
Ser	AGU *	0.24	Pro	CCU ***	0.97	Thr	ACU ***	0.57	Pro	CCU ***	0.84	Leu	UUA ***	0.97
Ser	UCU ***	1.25	Ser	AGU *	0.14	Val	GUA *	0.24	Ser	AGU **	0.43	Pro	CCU ***	0.84
Thr	ACA *	0.12	Ser	UCU ***	0.96				Ser	UCU ***	0.78	Ser	AGU *	0.18
Thr	ACU ***	0.78	Thr	ACA *	0.24				Thr	ACA *	0.29	Ser	UCU ***	0.78
Val	GUU **	0.39	Thr	ACU ***	0.55				Thr	ACU **	0.3	Thr	ACU *	0.1
			Val	GUA *	0.26				Tyr	UAU *	0.08	Tyr	UAU *	0.2
									Val	GUA *	0.17	Val	GUA ***	0.65

Note: * indicates ΔRSCU ≥ 0.08, ** indicates ΔRSCU ≥ 0.3, and *** indicates ΔRSCU ≥ 0.5.

## Data Availability

The sequence data reported in this paper have been deposited in the Science Data Bank (https://www.scidb.cn/ (accessed on 3 July 2025)), and the CSTR is 31253.11.sciencedb.27462, publicly accessible at https://www.scidb.cn/s/IFZfmy (accessed on 3 July 2025). Data will be made available on request from the author via email (ygsn2705@126.com).

## Supplementary Materials

The following supporting information can be downloaded at https://www.mdpi.com/article/10.3390/ijms262110266/s1.
