# Supplementary material for: Comparative Genomics of Triticum, Secale, and Triticale: Codon Usage Bias in Chloroplast Genomes and Its Implications for Evolution and Genetic Engineering
Source: Int J Mol Sci. 2025 Oct 22;26(21):10266. doi: 10.3390/ijms262110266 (PMC12610814; doi:10.3390/ijms262110266)
Supplement: Supplementary file 1 [file ijms-26-10266-s001.zip › ijms-3837391-supplementary.pdf]

Table S1 Table 1. Summary of SCUO, MILC, GC, GC1, GC2 and GC3

**Species name**	**SCUO**	**MILC**	**GC**	**GC1**	**GC2**	**GC3**	**Regression coefficient of GC1 on GC3**	**Regression coefficient of GC2 on GC3**
× *Triticosecale sp.*	0.23	0.8339	38.91	46.83	39.62	30.28	0.24	-0.055
Triticum monococcum	0.22	0.8344	39.16	46.95	39.96	30.57	0.26	0.109
Triticum turgidum	0.23	0.8312	38.78	46.81	39.68	29.84	0.32	0.039
Triticum aestivum	0.22	0.8338	39.16	47.02	39.97	30.49	0.29	0.128
Secale cereale	0.22	0.8338	39.05	46.97	39.84	30.33	0.29	0.084

Table S2 Relative synonymous codon usage of cp genes in different species of Triticum, Secale and Triticale.

**Amino acids**	Codon	× *Triticosecale sp.*		*Triticum monococcum*		*Triticum turgidum*		*Triticum aestivum*		*Secale cereale*	
		No.	RSCU	No.	RSCU	No.	RSCU	No.	RSCU	No.	RSCU
Ala	GCG	113	0.4224	121	0.4428	155	0.5828	477	1.7616	155	0.5796
	GCA	327	1.2224	481	1.7604	324	1.218	328	1.2116	327	1.2224
	GCU	472	1.7644	160	0.5856	470	1.7668	119	0.4396	472	1.7644
	GCC	158	0.5908	331	1.2112	115	0.4324	159	0.5872	116	0.4336
Cys	UGC	44	0.4916	45	0.4838	135	1.5084	138	1.5	139	1.5028
	UGU	135	1.5084	141	1.5162	44	0.4916	46	0.5	46	0.4972
Asp	GAC	138	0.432	508	1.5558	137	0.4384	498	1.5514	491	1.544
	GAU	501	1.568	145	0.4442	488	1.5616	144	0.4486	145	0.456
Glu	GAG	244	0.5264	699	1.4856	238	0.5214	244	0.5248	685	1.4794
	GAA	683	1.4736	242	0.5144	675	1.4786	686	1.4752	241	0.5206
Phe	UUC	322	0.668	331	0.6748	639	1.351	318	0.657	645	1.3298
	UUU	642	1.332	650	1.3252	307	0.649	650	1.343	325	0.6702
Gly	GGA	499	1.5668	505	1.5528	487	1.5508	230	0.716	225	0.7088
	GGG	227	0.7128	152	0.4672	221	0.704	152	0.4732	398	1.2536
	GGC	149	0.468	411	1.2636	400	1.274	405	1.2608	151	0.4756
	GGU	399	1.2528	233	0.7164	148	0.4712	498	1.55	496	1.5624
His	CAC	107	0.5336	296	1.4618	282	1.4612	108	0.5454	293	1.4686
	CAU	294	1.4664	109	0.5382	104	0.5388	288	1.4546	106	0.5314
Ile	AUA	436	0.9315	449	0.9387	699	1.5228	442	0.9417	444	0.942
	AUU	711	1.5192	725	1.5156	427	0.9303	707	1.5063	258	0.5475
	AUC	257	0.549	261	0.5457	251	0.5469	259	0.5517	712	1.5105
Lys	AAG	222	0.5224	225	0.5208	211	0.5122	216	0.5124	612	1.4836
	AAA	628	1.4776	639	1.4792	613	1.4878	627	1.4876	213	0.5164
Leu	CUC	126	0.405	94	0.2988	391	1.2912	91	0.294	631	2.0352
	UUA	634	2.0388	644	2.0466	338	1.116	131	0.423	399	1.287
	CUG	92	0.2958	264	0.8388	628	2.0736	261	0.8424	91	0.2934
	UUG	346	1.1124	347	1.1028	119	0.393	397	1.2816	266	0.858
	CUU	406	1.3056	407	1.2936	88	0.2904	636	2.0526	129	0.4164
	CUA	262	0.8424	132	0.4194	253	0.8352	343	1.107	344	1.1094
Asn	AAC	173	0.5044	175	0.5022	499	1.4896	175	0.511	168	0.4978
	AAU	513	1.4956	522	1.4978	171	0.5104	510	1.489	507	1.5022
Pro	CCU	288	1.5588	206	1.0884	194	1.0732	83	0.442	204	1.0968
	CCC	167	0.904	293	1.5484	286	1.5824	172	0.916	170	0.914
	CCA	203	1.0988	176	0.93	80	0.4428	201	1.0704	80	0.43
	CCG	81	0.4384	82	0.4332	163	0.9016	295	1.5712	290	1.5592
Gln	CAA	450	1.5332	459	1.5352	135	0.4632	140	0.4722	138	0.4686
	CAG	137	0.4668	139	0.4648	448	1.5368	453	1.5278	451	1.5314
Arg	AGG	115	0.672	98	0.5598	207	1.2612	216	1.2546	219	1.2984
	CGC	93	0.5436	234	1.3374	224	1.3644	303	1.7598	94	0.5574
	CGG	74	0.4326	304	1.737	66	0.402	230	1.3362	292	1.731
	AGA	297	1.7352	221	1.263	106	0.6456	71	0.4122	225	1.3338
	CGU	230	1.344	73	0.417	289	1.7604	96	0.5574	114	0.6756
	CGA	218	1.2738	120	0.6858	93	0.5664	117	0.6798	68	0.4032
Ser	UCU	343	1.6638	347	1.6512	82	0.4098	343	1.6704	83	0.4032
	AGU	257	1.2468	240	1.1418	225	1.125	210	1.023	236	1.1466
	UCA	211	1.0236	212	1.0086	205	1.0248	258	1.2564	207	1.0056
	AGC	85	0.4122	88	0.4188	253	1.2648	84	0.4092	106	0.5148
	UCC	236	1.1448	264	1.2564	334	1.6698	235	1.1442	258	1.2534
	UCG	105	0.5094	110	0.5232	101	0.5052	102	0.4968	345	1.6764
Thr	ACC	157	0.69	109	0.4728	387	1.7432	394	1.7396	397	1.7548
	ACU	396	1.7408	257	1.1148	98	0.4416	253	1.1168	105	0.464
	ACA	254	1.1164	156	0.6768	251	1.1308	104	0.4592	153	0.6764
	ACG	103	0.4528	400	1.7352	152	0.6848	155	0.6844	250	1.1048
Val	GUC	120	0.4844	125	0.4964	373	1.5348	121	0.4844	124	0.5068
	GUU	365	1.4732	387	1.5372	362	1.4896	126	0.5044	363	1.4832
	GUA	382	1.542	370	1.4696	120	0.494	369	1.4776	373	1.524
	GUG	124	0.5004	125	0.4964	117	0.4816	383	1.5336	119	0.4864
Tyr	UAC	133	0.415	511	1.5844	498	1.591	506	1.5912	507	1.5894
	UAU	508	1.585	134	0.4156	128	0.409	130	0.4088	131	0.4106

Table S3 The MILC of *Triticum, Secale and Triticale*

× *Triticosecale sp.*		*Triticum monococcum*		*Triticum turgidum*		*Triticum aestivum*		*Secale cereale*	
**gene**	**MILC**	**gene**	**MILC**	**gene**	**MILC**	**gene**	**MILC**	**gene**	**MILC**
atpA	0.814	atpA	0.815	atpA	0.814	atpA	0.814	atpA	0.813
atpB	0.807	atpB	0.808	atpB	0.807	atpB	0.808	atpB	0.806
atpE	0.824	atpE	0.825	atpE	0.824	atpE	0.824	atpE	0.822
atpF	0.841	atpF	0.841	atpF	0.841	atpF	0.841	atpF	0.841
atpI	0.835	atpI	0.836	atpI	0.835	atpI	0.835	atpI	0.834
ccsA	0.863	ccsA	0.859	ccsA	0.863	ccsA	0.863	ccsA	0.861
cemA	0.828	cemA	0.83	cemA	0.828	cemA	0.828	cemA	0.829
clpP	0.825	clpP	0.823	clpP	0.825	clpP	0.825	clpP	0.823
infA	0.84	infA	0.84	matK	0.827	matK	0.827	matK	0.827
matK	0.827	matK	0.827	ndhA	0.858	ndhA	0.858	ndhA	0.86
ndhA	0.859	ndhA	0.859	ndhB	0.844	ndhB	0.844	ndhB	0.845
ndhB	0.844	ndhB	0.845	ndhC	0.829	ndhC	0.829	ndhC	0.828
ndhC	0.829	ndhC	0.829	ndhD	0.865	ndhD	0.865	ndhD	0.864
ndhD	0.865	ndhD	0.865	ndhE	0.864	ndhE	0.864	ndhE	0.864
ndhE	0.864	ndhE	0.864	ndhF	0.856	ndhF	0.856	ndhF	0.858
ndhF	0.856	ndhF	0.857	ndhG	0.873	ndhG	0.873	ndhG	0.872
ndhG	0.873	ndhG	0.873	ndhH	0.85	ndhH	0.85	ndhH	0.853
ndhH	0.85	ndhH	0.85	ndhI	0.824	ndhI	0.824	ndhI	0.823
ndhI	0.824	ndhI	0.823	ndhJ	0.838	ndhJ	0.838	ndhJ	0.837
ndhJ	0.838	ndhJ	0.838	ndhK	0.847	ndhK	0.847	ndhK	0.856
ndhK	0.847	ndhK	0.845	petA	0.838	petA	0.838	petA	0.838
petA	0.838	petA	0.839	petB	0.813	petB	0.813	petB	0.818
petB	0.814	petB	0.816	petD	0.84	petD	0.84	petD	0.839
petD	0.837	petD	0.839	psaA	0.807	psaA	0.807	psaA	0.806
psaA	0.807	psaA	0.806	psaB	0.831	psaB	0.831	psaB	0.831
psaB	0.831	psaB	0.831	psbA	0.666	psbA	0.666	psbA	0.667
psbA	0.666	psbA	0.666	psbB	0.804	psbB	0.804	psbB	0.805
psbB	0.804	psbB	0.804	psbC	0.811	psbC	0.811	psbC	0.812
psbC	0.811	psbC	0.808	psbD	0.766	psbD	0.766	psbD	0.767
psbD	0.766	psbD	0.765	rbcL	0.721	rbcL	0.721	rbcL	0.721
rbcL	0.721	rbcL	0.721	rpl14	0.823	rpl14	0.823	rpl14	0.816
rpl14	0.823	rpl14	0.816	rpl16	0.878	rpl16	0.878	rpl16	0.878
rpl16	0.882	rpl16	0.87	rpl2	0.839	rpl2	0.839	rpl22	0.833
rpl20	0.871	rpl2	0.848	rpl20	0.871	rpl20	0.871	rpoA	0.844
rpl22	0.835	rpl20	0.871	rpl22	0.835	rpl22	0.835	rpoB	0.844
rpoA	0.842	rpl22	0.833	rpoA	0.842	rpoA	0.842	rpoC1	0.847
rpoB	0.843	rpoA	0.842	rpoB	0.843	rpoB	0.844	rpoC2	0.841
rpoC1	0.846	rpoB	0.844	rpoC1	0.846	rpoC1	0.846	rps11	0.825
rpoC2	0.841	rpoC1	0.845	rpoC2	0.841	rpoC2	0.841	rps12	0.857
rps11	0.826	rpoC2	0.842	rps11	0.826	rps11	0.826	rps14	0.857
rps12	0.854	rps11	0.826	rps14	0.854	rps12	0.855	rps18	0.861
rps14	0.854	rps12	0.857	rps18	0.857	rps14	0.854	rps2	0.835
rps18	0.857	rps14	0.857	rps2	0.836	rps18	0.857	rps3	0.803
rps2	0.836	rps18	0.859	rps3	0.803	rps2	0.836	rps4	0.84
rps3	0.803	rps2	0.836	rps4	0.841	rps3	0.802	rps7	0.836
rps4	0.841	rps3	0.799	rps7	0.836	rps4	0.841	rps8	0.884
rps7	0.836	rps4	0.841	rps8	0.884	rps7	0.836	ycf15	0.888
rps8	0.884	rps7	0.836	ycf3	0.834	rps8	0.884	ycf2	0.864
ycf2	0.864	rps8	0.883	ycf4	0.832	ycf2	0.888	ycf3	0.834
ycf3	0.862	ycf15	0.89			ycf3	0.834	ycf4	0.831
ycf4	0.832	ycf2	0.864			ycf4	0.832	ycf68	0.884
ycf68	0.884	ycf3	0.834			ycf68	0.884		
		ycf4	0.832						
		ycf68	0.884						
